# 3-Carb­oxy­methyl-1*H*-indole-4-carb­oxy­lic acid

**DOI:** 10.1107/S1600536811051865

**Published:** 2011-12-17

**Authors:** Shulin Mao

**Affiliations:** a Ordered Matter Science Research Center, College of Chemistry and Chemical Engineering, Southeast University, Nanjing 211189, People’s Republic of China

## Abstract

In the title compound, C_11_H_9_NO_4_, the carboxyl group bonded to the six-membered ring lies close to the plane of the 1*H*-indole ring system [dihedral angle = 13.13 (9)°], whereas the carb­oxy­lic acid group linked to the five-membered ring by a methyl­ene bridge is close to perpendicular [78.85 (9)°]. In the crystal, O—H⋯O and N—H⋯O hydrogen bonds link the mol­ecules, generating (110) sheets.

## Related literature

For background to indoles as pharmaceuticals, see: Lang *et al.* (2011 ▶).
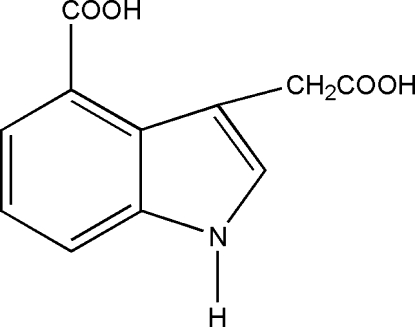


## Experimental

### 

#### Crystal data

C_11_H_9_NO_4_
*M* *_r_* = 219.19
Triclinic, 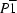

*a* = 5.4573 (11) Å
*b* = 9.823 (2) Å
*c* = 9.940 (2) Å
α = 73.90 (3)°
β = 78.57 (3)°
γ = 80.40 (3)°
*V* = 498.3 (2) Å^3^
*Z* = 2
Mo *K*α radiation
μ = 0.11 mm^−1^
*T* = 293 K
0.30 × 0.23 × 0.20 mm

#### Data collection

Rigaku SCXmini CCD diffractometer
Absorption correction: multi-scan (*CrystalClear*; Rigaku, 2005 ▶) *T*_min_ = 0.977, *T*_max_ = 0.984
5115 measured reflections
2263 independent reflections
1786 reflections with *I* > 2σ(*I*)
*R*_int_ = 0.028

#### Refinement

*R*[*F* ^2^ > 2σ(*F* ^2^)] = 0.046
*wR*(*F* ^2^) = 0.132
*S* = 0.95
2239 reflections
147 parameters
H-atom parameters constrained
Δρ_max_ = 0.30 e Å^−3^
Δρ_min_ = −0.21 e Å^−3^

### 

Data collection: *CrystalClear* (Rigaku, 2005 ▶); cell refinement: *CrystalClear*; data reduction: *CrystalClear*; program(s) used to solve structure: *SHELXS97* (Sheldrick, 2008 ▶); program(s) used to refine structure: *SHELXL97* (Sheldrick, 2008 ▶); molecular graphics: *DIAMOND* (Brandenburg & Putz, 2005 ▶); software used to prepare material for publication: *SHELXL97*.

## Supplementary Material

Crystal structure: contains datablock(s) I, global. DOI: 10.1107/S1600536811051865/hb6541sup1.cif

Structure factors: contains datablock(s) I. DOI: 10.1107/S1600536811051865/hb6541Isup2.hkl

Supplementary material file. DOI: 10.1107/S1600536811051865/hb6541Isup3.cml

Additional supplementary materials:  crystallographic information; 3D view; checkCIF report

## Figures and Tables

**Table 1:** Hydrogen-bond geometry (Å, °)

*D*—H⋯*A*	*D*—H	H⋯*A*	*D*⋯*A*	*D*—H⋯*A*
O1—H1⋯O2^i^	0.82	1.83	2.6504 (19)	174
O4—H4⋯O3^ii^	0.82	1.91	2.719 (2)	171
N1—H1*B*⋯O3^iii^	0.86	2.38	3.152 (2)	150

Symmetry codes: (i) 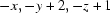; (ii) 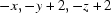; (iii) 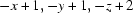.

**Fig. 1.**
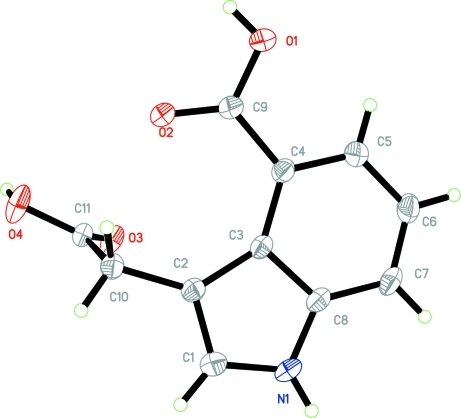
The asymmetric unit of (I) with displacement ellipsoids drawn at the 30% probability level.

**Fig. 2.**
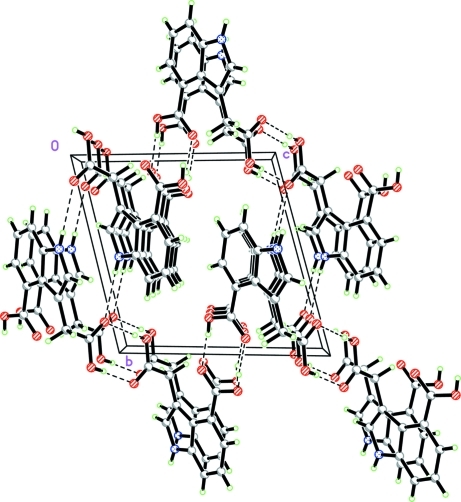
Packing diagram.

## supplementary crystallographic information

### Experimental

To a solution of the title compound (0.2 g) in methanol (20 ml) by stirred at room temperature and then placed in a dark place. Yellow single crystals were obtained by slow evaporation of the solution over a period of 5 days.

### Refinement

Positional parameters of all the H atoms were calculated geometrically and refined using a riding model,, with C—H = 0.94Å and *U*_iso_(H) =1.2Ueq(C) respectively.

### Crystal data

**Table:**

C_11_H_9_NO_4_	*V* = 498.3 (2) Å^3^
*M**_r_* = 219.19	*Z* = 2
Triclinic, *P*1	*F*(000) = 228
Hall symbol: -P 1	*D*_x_ = 1.461 Mg m^−^^3^
*a* = 5.4573 (11) Å	Mo *K*α radiation, λ = 0.71073 Å
*b* = 9.823 (2) Å	θ = 3.4–27.4°
*c* = 9.940 (2) Å	µ = 0.11 mm^−^^1^
α = 73.90 (3)°	*T* = 293 K
β = 78.57 (3)°	Prism, yellow
γ = 80.40 (3)°	0.30 × 0.23 × 0.20 mm

### Data collection

**Table:**

Rigaku SCXmini CCD diffractometer	2263 independent reflections
Radiation source: fine-focus sealed tube	1786 reflections with *I* > 2σ(*I*)
graphite	*R*_int_ = 0.028
Detector resolution: 13.6612 pixels mm^-1^	θ_max_ = 27.4°, θ_min_ = 3.4°
CCD_Profile_fitting scans	*h* = −6→7
Absorption correction: multi-scan (*CrystalClear*; Rigaku, 2005)	*k* = −12→12
*T*_min_ = 0.977, *T*_max_ = 0.984	*l* = −12→12
5115 measured reflections	

### Refinement

**Table:**

Refinement on *F*^2^	Primary atom site location: structure-invariant direct methods
Least-squares matrix: full	Secondary atom site location: difference Fourier map
*R*[*F*^2^ > 2σ(*F*^2^)] = 0.046	Hydrogen site location: inferred from neighbouring sites
*wR*(*F*^2^) = 0.132	H-atom parameters constrained
*S* = 0.95	*w* = 1/[σ^2^(*F*_o_^2^) + (0.0712*P*)^2^ + 0.1712*P*] where *P* = (*F*_o_^2^ + 2*F*_c_^2^)/3
2239 reflections	(Δ/σ)_max_ = 0.015
147 parameters	Δρ_max_ = 0.30 e Å^−^^3^
0 restraints	Δρ_min_ = −0.21 e Å^−^^3^

### Special details

**Table:**

Geometry. All e.s.d.'s (except the e.s.d. in the dihedral angle between two l.s. planes) are estimated using the full covariance matrix. The cell e.s.d.'s are taken into account individually in the estimation of e.s.d.'s in distances, angles and torsion angles; correlations between e.s.d.'s in cell parameters are only used when they are defined by crystal symmetry. An approximate (isotropic) treatment of cell e.s.d.'s is used for estimating e.s.d.'s involving l.s. planes.
Refinement. Refinement of *F*^2^ against ALL reflections. The weighted *R*-factor *wR* and goodness of fit *S* are based on *F*^2^, conventional *R*-factors *R* are based on *F*, with *F* set to zero for negative *F*^2^. The threshold expression of *F*^2^ > σ(*F*^2^) is used only for calculating *R*-factors(gt) *etc*. and is not relevant to the choice of reflections for refinement. *R*-factors based on *F*^2^ are statistically about twice as large as those based on *F*, and *R*- factors based on ALL data will be even larger.

### Fractional atomic coordinates and isotropic or equivalent isotropic displacement parameters (Å^2^)

**Table:**

	*x*	*y*	*z*	*U*_iso_*/*U*_eq_	
O3	0.1544 (2)	0.84288 (12)	0.97213 (13)	0.0443 (3)	
O2	0.1686 (3)	0.91438 (13)	0.62549 (15)	0.0579 (4)	
O1	0.0340 (3)	0.82135 (14)	0.47736 (16)	0.0648 (5)	
H1	−0.0320	0.9036	0.4520	0.097*	
O4	0.2593 (3)	1.06204 (12)	0.86103 (17)	0.0588 (4)	
H4	0.1255	1.0856	0.9079	0.088*	
N1	0.7240 (3)	0.48127 (14)	0.87277 (16)	0.0423 (4)	
H1B	0.8103	0.4047	0.9134	0.051*	
C3	0.4527 (3)	0.63513 (15)	0.73814 (15)	0.0300 (3)	
C11	0.2985 (3)	0.92105 (16)	0.88625 (17)	0.0357 (4)	
C9	0.1571 (3)	0.81052 (17)	0.58232 (17)	0.0375 (4)	
C4	0.2738 (3)	0.66441 (16)	0.64359 (16)	0.0337 (4)	
C2	0.5741 (3)	0.71447 (16)	0.80476 (17)	0.0330 (3)	
C8	0.5522 (3)	0.48888 (16)	0.78546 (17)	0.0347 (4)	
C7	0.4775 (3)	0.37626 (17)	0.74825 (19)	0.0416 (4)	
H7	0.5467	0.2826	0.7820	0.050*	
C5	0.2020 (4)	0.55078 (18)	0.60716 (19)	0.0440 (4)	
H5	0.0857	0.5703	0.5454	0.053*	
C1	0.7343 (3)	0.61564 (19)	0.8840 (2)	0.0423 (4)	
H1A	0.8362	0.6374	0.9380	0.051*	
C10	0.5422 (3)	0.87055 (16)	0.80186 (19)	0.0375 (4)	
H10A	0.5509	0.9260	0.7040	0.045*	
H10B	0.6816	0.8894	0.8385	0.045*	
C6	0.2990 (4)	0.40813 (18)	0.6605 (2)	0.0463 (4)	
H6	0.2424	0.3352	0.6363	0.056*	

### Atomic displacement parameters (Å^2^)

**Table:**

	*U*^11^	*U*^22^	*U*^33^	*U*^12^	*U*^13^	*U*^23^
O3	0.0485 (7)	0.0270 (6)	0.0508 (7)	0.0021 (5)	−0.0007 (6)	−0.0082 (5)
O2	0.0848 (10)	0.0310 (6)	0.0679 (9)	0.0111 (6)	−0.0480 (8)	−0.0141 (6)
O1	0.1008 (12)	0.0354 (7)	0.0696 (9)	0.0118 (7)	−0.0576 (9)	−0.0129 (6)
O4	0.0616 (9)	0.0258 (6)	0.0804 (10)	−0.0021 (6)	0.0096 (7)	−0.0159 (6)
N1	0.0411 (8)	0.0319 (7)	0.0511 (8)	0.0107 (6)	−0.0162 (7)	−0.0083 (6)
C3	0.0320 (7)	0.0255 (7)	0.0294 (7)	0.0008 (6)	−0.0016 (6)	−0.0068 (5)
C11	0.0407 (9)	0.0251 (7)	0.0438 (9)	0.0001 (6)	−0.0134 (7)	−0.0106 (6)
C9	0.0443 (9)	0.0319 (8)	0.0362 (8)	0.0010 (7)	−0.0138 (7)	−0.0064 (6)
C4	0.0388 (8)	0.0274 (7)	0.0333 (8)	−0.0005 (6)	−0.0069 (6)	−0.0061 (6)
C2	0.0302 (7)	0.0313 (8)	0.0372 (8)	0.0007 (6)	−0.0056 (6)	−0.0105 (6)
C8	0.0355 (8)	0.0286 (8)	0.0354 (8)	0.0043 (6)	−0.0041 (6)	−0.0063 (6)
C7	0.0517 (10)	0.0234 (7)	0.0452 (9)	0.0025 (7)	−0.0043 (8)	−0.0076 (6)
C5	0.0546 (10)	0.0338 (8)	0.0481 (10)	−0.0030 (7)	−0.0194 (8)	−0.0112 (7)
C1	0.0378 (9)	0.0411 (9)	0.0494 (10)	0.0038 (7)	−0.0144 (7)	−0.0138 (7)
C10	0.0366 (8)	0.0313 (8)	0.0470 (9)	−0.0034 (6)	−0.0101 (7)	−0.0122 (7)
C6	0.0628 (12)	0.0300 (8)	0.0498 (10)	−0.0063 (8)	−0.0128 (9)	−0.0128 (7)

### Geometric parameters (Å, °)

**Table:**

O3—C11	1.228 (2)	C9—C4	1.483 (2)
O2—C9	1.226 (2)	C4—C5	1.399 (2)
O1—C9	1.321 (2)	C2—C1	1.376 (2)
O1—H1	0.8200	C2—C10	1.507 (2)
O4—C11	1.3255 (19)	C8—C7	1.401 (2)
O4—H4	0.8200	C7—C6	1.375 (3)
N1—C1	1.366 (2)	C7—H7	0.9300
N1—C8	1.379 (2)	C5—C6	1.406 (2)
N1—H1B	0.8600	C5—H5	0.9300
C3—C4	1.428 (2)	C1—H1A	0.9300
C3—C8	1.430 (2)	C10—H10A	0.9700
C3—C2	1.455 (2)	C10—H10B	0.9700
C11—C10	1.512 (2)	C6—H6	0.9300
			
C9—O1—H1	109.5	N1—C8—C3	108.27 (14)
C11—O4—H4	109.5	C7—C8—C3	123.86 (15)
C1—N1—C8	108.65 (13)	C6—C7—C8	118.15 (15)
C1—N1—H1B	125.7	C6—C7—H7	120.9
C8—N1—H1B	125.7	C8—C7—H7	120.9
C4—C3—C8	116.31 (14)	C4—C5—C6	122.44 (16)
C4—C3—C2	137.91 (14)	C4—C5—H5	118.8
C8—C3—C2	105.78 (13)	C6—C5—H5	118.8
O3—C11—O4	122.70 (15)	N1—C1—C2	111.03 (15)
O3—C11—C10	125.15 (14)	N1—C1—H1A	124.5
O4—C11—C10	112.12 (14)	C2—C1—H1A	124.5
O2—C9—O1	121.54 (15)	C2—C10—C11	114.51 (14)
O2—C9—C4	123.59 (15)	C2—C10—H10A	108.6
O1—C9—C4	114.86 (14)	C11—C10—H10A	108.6
C5—C4—C3	119.06 (14)	C2—C10—H10B	108.6
C5—C4—C9	117.85 (15)	C11—C10—H10B	108.6
C3—C4—C9	123.09 (14)	H10A—C10—H10B	107.6
C1—C2—C3	106.26 (14)	C7—C6—C5	120.11 (16)
C1—C2—C10	122.09 (15)	C7—C6—H6	119.9
C3—C2—C10	131.61 (14)	C5—C6—H6	119.9
N1—C8—C7	127.86 (15)		

### Hydrogen-bond geometry (Å, °)

**Table:**

*D*—H···*A*	*D*—H	H···*A*	*D*···*A*	*D*—H···*A*
O1—H1···O2^i^	0.82	1.83	2.6504 (19)	174
O4—H4···O3^ii^	0.82	1.91	2.719 (2)	171
N1—H1B···O3^iii^	0.86	2.38	3.152 (2)	150

Symmetry codes: (i) −*x*, −*y*+2, −*z*+1; (ii) −*x*, −*y*+2, −*z*+2; (iii) −*x*+1, −*y*+1, −*z*+2.
